# Toxicity of nanomaterials; an undermined issue

**DOI:** 10.1186/s40199-014-0059-4

**Published:** 2014-08-15

**Authors:** Mehdi Mogharabi, Mohammad Abdollahi, Mohammad Ali Faramarzi

**Affiliations:** Department of Pharmaceutical Biotechnology, Faculty of Pharmacy & Biotechnology Research Center, Tehran University of Medical Sciences, Tehran, 1417614411 Iran; Department of Toxicology and Pharmacology, Faculty of Pharmacy and Pharmaceutical Sciences Research Center, Tehran University of Medical Sciences, Tehran, 1417614411 Iran

**Keywords:** Adverse health effects, Drug delivery, Nanomaterials, Nanomedicine, Toxicity

## Abstract

Nanomaterials are employed in extensive variety of commercial products such as electronic components, cosmetics, food, sports equipment, biomedical applications, and medicine. With the increasing utilization of engineered nanomaterials, the potential exposure of human to nanoparticles is rapidly increasing. Nowadays when new nanomaterials with new applications are introduced, mostly good and positive effects are mentioned whereas possible hazards arising from nanosize of the compounds are undermined. Toxicology studies of nanomaterials demonstrate some adverse effects in some human organs such as central nerve system, immune system, and lung. There is lack of complete information about human toxicity and environmental waste of nanomaterials. We aimed to highlight current toxicological concerns of potentially useful nanomaterials which are now used in pharmaceutical and biomedical sciences.

Nanotechnology possesses a progressively sophisticated ability to manipulate matter at the nanoscale, making new materials, products, and devices that demonstrate new and unique behavior. Despite this explosive growth, the potential safety risks associated with consumer products containing nanoscale materials are still being learned. Exposure to products containing nanomaterials may cause effects which differ from those observed with conventionally scaled materials. Nowadays when new nanomaterials with new applications are introduced, mostly good and positive effects are mentioned whereas possible hazards arising from nanosize of the compounds are undermined. In addition to the chance of direct exposure to nanocompounds if used in human as a medicine, there are concerns on exposure during product manufacturing or through waste sources. Development of nanomaterials with numerous industrial applications such as electronic components, medical devices, tires, food and beverage, pharmaceuticals, and cosmetics has increased the chance of accumulation in the environment and consequent entering into food chain and human body (Figure 1) [1,2]. As an example, Australian government surprisingly reported that 30% of zinc sunscreens and 70% of titanium sunscreens are now formulated with nanosized ingredients (http://www.tga.gov.au/; accessed June 17, 2014). Also, several new products containing nanoparticles are being launched by food industries with the understanding to improve appearance, efficiency, and packaging of foods [3]. For instance, ZnO and MgO nanoparticles due to their antibacterial activities are used as the additive to polymer matrix of food packing [4]. On the other hand, there has been progressive interest in the pharmaceutical companies to use nanomaterials with the aim to better drug delivery. In this way, some drugs have got approval for use in human such as Doxil® known as one of several nanoparticle formulations of doxorubicin or Abraxane® that is an albumin-based nanoparticle formulation of paclitaxel used to treat different kinds of cancers and acquired immunodeficiency syndrome. Poly ethylene glycol (PEG)-linked proteins also represent an important group of therapeutic macromolecules that have been widely investigated within the context of nanoparticles. Covalent attachment of PEG to proteins has been demonstrated to prolong their circulation due to the size of molecule and reduced kidney clearance. Oncospar®, Pegasys®, and Neulasta® are approved by FDA as PEGylated protein for acute lymphoblastic leukemia, hepatitis C, and neutropenia, respectively. Macugen® (Pegaptanib) is an approved nanoscaled formulation of aptamer applied as an antagonist to vascular endothelial growth factor that lessens growth of the vessels located within the eye (Table 1) [5]. Besides the growth in applications of nanomaterials in various aspects of human life, concerns on potential adverse human health effects of nanoparticles are escalating. Intravenous and subcutaneous injections of nanomaterial-based carriers delivers exogenous nanoparticles into the body with the aim to better distribution whereas broader spread may cause toxicity and undesirable interaction with biological macromolecules. Injected nanomaterials smaller than 100 nm are efficiently transported via interstitial flow to the draining lymphatics and lymph nodes. Meanwhile, they can reach most of organs dependent on the size of particle and surface characteristics. Besides injection, other routs of exposure like dermal, inhalation, and ingestion are also common. The penetration to skin by use of cosmetics with nanoparticles is the most relevant dermal route of exposure in human. Studies show that metallic nanoparticles smaller than 10 nm are able to penetrate the epidermal layers [1,6]. Larese et al. [7] demonstrated the permeation of silver nanoparticles through the stratum corneum of damaged skin in an in vitro diffusion cell system. Nanoparticles possess the ability to induce lung inflammation by not only stimulating pulmonary epithelial cells to generate proinflammatory cytokines, but also via causing endothelial cells to produce leukocyte adhesion molecules and circulating leukocytes [8]. Non-surprisingly destructive effects of metal containing nanomaterials on the brain vasculature have grown the toxicity concerns. For instance, it has been demonstrated that silver nanoparticles disrupt blood brain barrier and cause neuronal degeneration [9]. A major challenge in drug delivery is to improve selective targeting and safe strategies but major caution should be made in especial group of patients like pregnant women, infants, and aged people. Giving examples about pregnant group, studies have shown that nanoparticles can easily cross the placental barrier and induce pregnancy complications [10] that is a serious matter. A toxicology study carried out on pregnant mice investigated the effect of fullerene solubilized with poly(vinylpyrrolidone) in water when injected intraperitoneally to pregnant mice at varying concentrations. The results demonstrated that fullerene nanoparticles were traced in the embryos and at higher doses caused significant toxicity and death [11]. In addition, titanium oxide nanoparticles, which are widely used in consumer products, possess the ability to transfer from pregnant mice to their offspring affecting the central nerve and genital systems [12].

**Figure 1:**
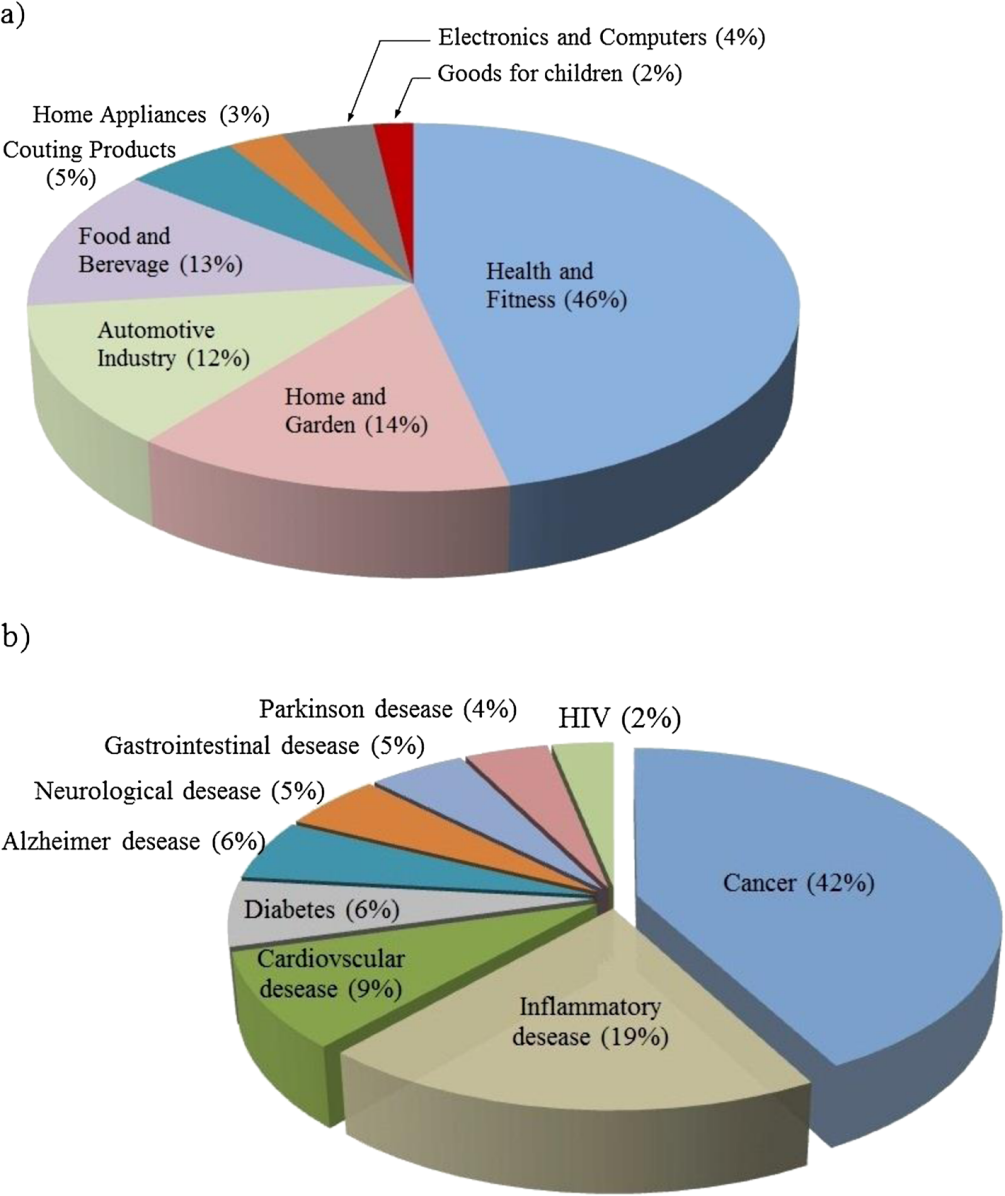
**Percent of various consumer products containing nanomaterials (a), percentage of articles published in scientific journals addressing the application of nanoparticles in various diseases (b), based on data obtained from Consumer Products Inventory [** http://www.nanotechproject.org/ **] and Scopus (accessed June 17, 2014).**

**Table 1:** **Some of clinically available nanoparticle containing drugs**

**Trade name**	**Nanomaterial**	**Company**	**Application**
Abraxane	Albumin	Abraxis Oncology	Cancer treatment
Basulin	Polyglutamate polymer dotted with insulin	Advanced Magnetics	Type I diabetes
Biovant	Nanosized calcium phosphate	BioSante Pharmaceuticals	Vaccine component
Combidex	Iron oxide nanoparticles	Advanced Magnetics	Tumor imaging
Doxil	PEGylated liposome	Orthobiotech	Cancer treatment
Oncospar	PEGylated asparaginase	Medac GmbH	Leukemia treatment
Opaxio™	Polyglutamic acid	Cell Therapeutics, Inc.	Cancer treatment

Using nanoparticles in medicine and drug delivery is growing rapidly while there are still non-resolved concerns about their risk of toxicity. It should not be forgotten that every material with smaller size can cross the cell membrane easier than materials with normal size. As a matter of fact, most of information about kinetics of materials come from tests of materials in the normal size and non-surprisingly there is a lack of data about kinetics of nanosized materials that has a major role in toxicity [13,14]. Graphene is a good example of a new nanomaterial that has raised new toxicological concerns [2]. Concerns on the use of nanomaterials also back to their waste in the environment. At the moment there is lack of information about pattern of accumulation of nanomaterials waste and their possible entrance into food chain. Here the facility of in-silico toxicology should come into help [15]. Therefore, scientists need a databank of biological effects, toxicity, biokinetics, as well as structure and molecular size of nanomaterials to be able to predict their toxicity. In other words, current need is to estimate different physical and chemical properties of the nanomaterials relevant to toxicity, environmental fate, and transport.
